# The fellowship of the Grignard: 21st century computational tools for hundred-year-old chemistry

**DOI:** 10.1039/d5sc01078k

**Published:** 2025-04-11

**Authors:** Michele Cascella, Sigbjørn Løland Bore, Odile Eisenstein

**Affiliations:** a Department of Chemistry and Hylleraas Centre for Quantum Molecular Sciences, University of Oslo PO Box 1033 Blindern 0315 Oslo Norway michele.cascella@kjemi.uio.no; b ICGM, Univ. Montpellier, CNRS, ENSCM Montpellier 34293 France odile.eisenstein@umontpellier.fr

## Abstract

This perspective begins with the discovery of the Grignard reaction by a graduate student in the last years of the 19th century, followed by describing why it has remained largely unexplained for more than a century. From the summary of what has been achieved, focusing on the computational aspects, it is now clear that further studies of the chemistry of any chemical species that is highly sensitive to solvents, such as Group I and II elements, require a holistic approach that includes the solute and the solvent together. *Ab initio* molecular dynamics, which meets these requirements, has produced some results but has hit hard limits due to its relatively high computational costs. In these days, it is becoming clear that data-driven methods, including machine learning potentials and simulations driven by quantitative on-the-fly calculation of relevant observables, have the potential to better and more completely explore the very large chemical space associated with the presence of a large number of species in solution. These methodologies have the chance to give the keys to enter the challenging and still poorly explored world of chemical species whose behaviour and reactivity are strongly influenced by the solvent and the experimental conditions.

## Abstract 2

The Grignard reaction remained unexplained for more than a century. Today, combining physics-based with machine-learning protocols, it is possible to study and understand the complex solution chemistry of main-group organometallic compounds.

## Graphical abstract

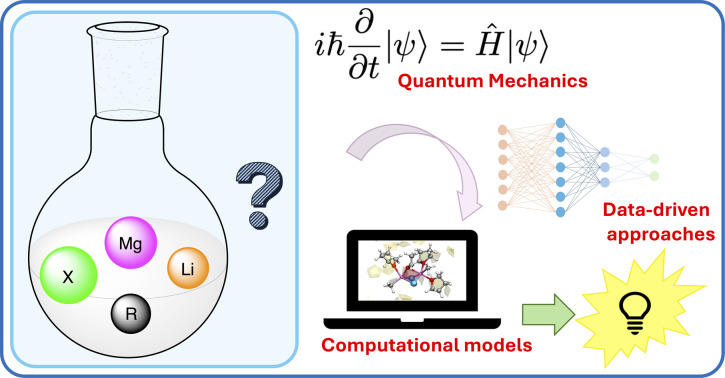

## 1 Victor Grignard and his discovery

### 1.1 Chemistry was not his first interest

In December 1894, Victor Grignard (1871–1935, Fig. 1) took up the post of a secondary technical assistant in the General Chemistry Department of the University of Lyon. At the beginning of the academic year 1895, he was promoted to a technical assistant in Philippe Barbier's laboratory and also began to work on his doctorate. Chemistry had not been his first love!^1,2^

**Fig. 1:**
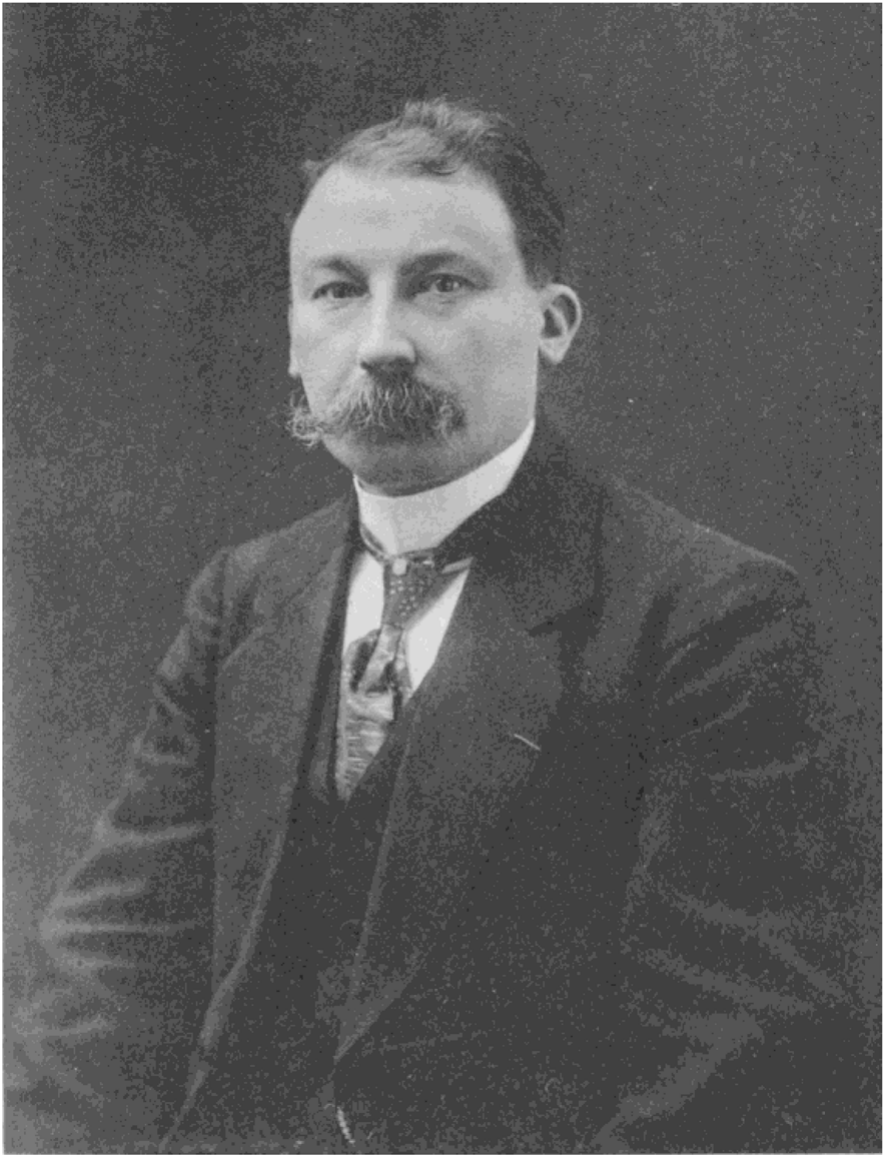
Victor Grignard in 1912. Fotograv. - Generalstabens Litografiska Anstalt Stockholm.

Being the son of a technician in the naval industry in Cherbourg, he was primarily interested in mathematics. He was in line to begin studies at École Normale Supérieure de Paris, but his scholarship was cancelled as the city ran out of funds after spending for the 1889 ‘Exposition Universelle’. He had to change his plans and was accepted into the École Normale Supérieure de Cluny, which trained teachers for vocational schools, but this ‘École’ closed after a general reorganisation of the country secondary schools. Grignard, who still had a year left of his scholarship, was enrolled as a mathematics student at the nearby University of Lyon. He got his BA in mathematics only on the second attempt and then started looking for a job. The exact details are unknown (need for a job or the good influence of a schoolmate), but he took the job of ‘préparateur adjoint’ at the Department of General Chemistry. Although he did not like chemistry at first, he was fortunate enough to be able to work for a year with the not-much-older Louis Bouvault (1864–1909), who completely overturned Grignard's prejudice against chemistry.

### 1.2 The discovery of the reaction

**Fig. 2:**
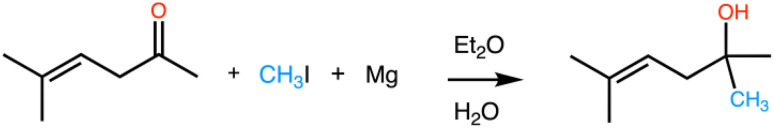
The reaction run by Philippe Barbier, which, as studied by Victor Grignard, led to the so-called Grignard reaction.

Philippe Barbier was a synthetic organic chemist with an open and modern mind, as evidenced by his early acceptance of the atomic theory. Barbier was developing the synthesis of organic compounds related to natural oils and considering reactions shown in Fig. 2. Barbier wanted to use magnesium because he expected it to be more reactive than zinc, which was used by Saytzeff to obtain tertiary alcohols from ketones.^3^ In fact, with all the ingredients shown in Fig. 2 added together, the reaction gave the expected alcohol, even though it was violent, difficult to control, and had a low yield. Consequently, Barbier published a note in the Comptes-Rendus de l’Académie des Sciences, stating that he had established for the first time that Zn could be replaced by Mg in the Saytzeff reaction and that this modification had enabled him to carry out some synthesis.^4^

Even if he claimed that he wanted to study this reaction further, he did not seem to pursue this line of research, suggesting it to Grignard as a topic for his doctorate thesis. After several unsuccessful trials, Grignard decided to try to identify an intermediate of the reaction. He discovered that the addition of the alkyl halide to Mg in diethyl ether led to a clear solution that did not spontaneously catch fire in the air but reacted rapidly with the added carbonyl substrate to yield, after hydrolysis, an alcohol. The reaction was considerably less violent than the first attempt by Barbier, gave reasonable yields, and was easier to control. Furthermore, the scope of the reaction was large, which implied that it was general and thus synthetically useful.

Grignard wrote a three-page single author note to the Comptes-Rendus de l’ Académie des Sciences entitled: “Sur quelques nouvelles combinaisons organométalliques du magnésium et des applications à des synthèses d'alcools et d'hydrocarbures” (On some new organometallic combinations with magnesium and their applications for the synthesis of alcohols and hydrocarbons). Following tradition, the note was presented at the Académie des Sciences by Henri Moissan (1906 Nobel Laureate for the discovery of fluorine) and was accepted for publication.^5^ The Grignard reaction was born.

Grignard defended his PhD in 1901. In 1912, he shared the Nobel prize in chemistry with Paul Sabatier. The motivation for the Nobel prize awarded to Grignard was: “for the discovery of the so-called Grignard reagent, which in recent years has greatly advanced the progress of organic chemistry”. More details can be found in the two articles mentioned above,^1,2^ with, in particular, an interesting account of the personal relationship between Barbier and Grignard.

## 2 The Grignard reaction: Why so complicated?

The Grignard reaction is probably one of the best-known reactions in organic chemistry, one that is taught in all introductory courses of organic chemistry. It is widely used for making new C–C bonds, as the scope of the reaction includes a large variety of R groups, as well as a large diversity of unsaturated electrophilic substrates, among which aldehydes and ketones are the most common species. Its prevalence in the academic and industrial environments is illustrated by the revenue associated with the Grignard reagent of around $2.6 billion in 2016, which is expected to reach $4.2 billion in 2030.^6^

### 2.1 The Schlenk equilibrium and the quest to determine the structures of magnesium species in organic solution

Although widely used and studied, the reaction remains poorly understood. As mentioned by the late Dietmar Seyferth: “Generally written as RMgX, the Grignard reagents in ethereal solution are more complicated than this simple formula indicates”.^7^ This statement sums up the continuing difficulties in determining the structures of these species in solution and, thus, the inability to know the nature of the reactive systems. The simultaneous presence of several species is a direct consequence of what was discovered by Schlenk and Schlenk – father and son – that RMgX is in equilibrium with R_2_Mg and MgX_2_*via* an X/R exchange.^8^ This naturally suggests that polynuclear species (at least dimers), in which the exchange can occur, must also exist, at least as intermediates. Considerable effort has therefore been devoted to characterising forms of the Grignard species. A crystalline solid CH_3_MgI·(O(*n*C_5_H_11_)_2_)_2_ was isolated and identified as such by elemental analysis (Mg and I) in 1908,^9^ but one had to wait about 60 years for the first characterization of a single crystal of EtMgBr·(OEt_2_)_2_ by X-ray diffraction.^10,11^ The latter study revealed a Mg⋯Br weak intermolecular interaction. Interestingly, no such interaction was seen in PhMgBr·(OEt_2_)_2_.^12^ At about the same time, the dimeric dibromo-bridged species (NEt_3_)Mg(Et)(μ-Br_2_)Mg(Et)(NEt_3_) was isolated from an *n*-butyl ether solution and characterized.^13^ The isolation of this dimeric species was attributed to the smaller and, thus, more favorable steric environment on the metal compared to the situation in the di-solvated monomer. Aggregates of higher nuclearity such as tetrameric species were also characterized by X-ray diffraction a few years later.^14^ As pointed out by Seyferth, the key observation was that “the species that crystallizes from a Grignard reagent solution does not necessarily directly reflect what species are swimming around in the solution”.^7^ And, in fact, many other species were shown to be *swimming* in solution through the use of a variety of spectroscopic and physical methods, including *inter alia*, ebullioscopy,^15,16^ molecular weight determination,^17^ calorimetry,^18,19^ NMR and IR spectroscopy,^20,21^ large angle X-ray scattering LAXS,^22^ and EXAFS.^23^

It was found that the structures of the Grignard species were strongly dependent on their nature (R and X) and also on the experimental conditions (solvent, temperature, and concentration). Thus, for instance, monomeric, dimeric and higher oligomeric species were present depending on the ethereal solvent, such as the very frequently used diethyl ether (Et_2_O) or tetrahydrofuran (THF), the halogen used (often Cl and Br) and the organic group R (usually a hydrocarbyl group).^7,24^ The numerous forms adopted by the Grignard reagent led to a generalization of the Schlenk equilibrium, which involved species like RMgCl(THF)_*n*_, MgR_2_(μ-Cl)_2_Mg(THF)_5/4_ and RMg(μ-Cl)_3_Mg(THF)_5_.^25,26^

### 2.2 More tools, further study of structures

Years after these seminal early studies, the quest for the nature of the Grignard in solution continued, benefiting from the availability of novel methods. Thus, cold spray ionisation mass spectrometry (CSI-MS) revealed the presence of neutral and charged (cationic as well as anionic) species for MeMgCl in solution.^26^ However, further studies of the same system indicated that the intact organometallic cations themselves were not present and that the charged species resulted from in-source ion-molecule reactions.^27^ Some of these authors pursued this study, combining electrospray-ionisation (ESI) mass spectrometry, electrical conductivity measurement, NMR spectroscopy (including DOSY), and density functional theory (DFT) calculations. They also indicated the presence of charged species but attributed these findings to the particular concentration conditions in the droplets.^28^ Although these mass spectrometry experiments provided data for conditions that were significantly different from those used in a standard Grignard reaction, they clearly showed how these species adopt structures that are strongly influenced by the experimental conditions. Finally, as expected, the solvation itself depends on the solute. There is a general preference for the coordination of four ligands on Mg(ii), but higher solvations have been observed, thus enlarging the first coordination shell.^29^

### 2.3 Nature of the reaction: nucleophilic and SET

Even in the absence of detailed knowledge of the reactive species, chemists have attempted to determine the reaction mechanism. For many years, the Grignard reaction was considered to be a nucleophilic addition of an anion R^−^ to the positively charged carbonyl carbon. However, evidence for the reaction going through the formation of a radical species R· called the single electron transfer (SET) reaction, was presented in previous studies.^30–33^ This proposition rationalized the formation of organic products that could not originate from a Grignard addition of R^−^ to the carbonyl, such as the dimer *via* the C–C bond of the two carbonyl substrates. Recent experiments fully clarified this dichotomy. Thus, Woerpel *et al.* established that the addition to aliphatic ketones does not favour single electron transfer and prefers the nucleophilic addition pathway.^34^ The SET reaction can occur with conjugated ketones but is not the exclusive pathway. In the case of phenyl ketone, the SET pathway was dominant only with tertiary alkyl like *tert*-butyl as the R group in RMgX. With a more electron-poor substrate like pentafluorophenyl ketone, SET was observed even with the primary R group (but not when R = allyl), asserting a clear influence of the substrate on the preferred reaction pathway.^35^ More details can be found in several reviews.^7,24,36,37^

## 3 Early theoretical/computational studies

### 3.1 Gas phase calculations

Given the complexity of the experimental situation, it is not surprising that only a limited number of computational studies on the Grignard reagent and reaction have been carried out. Early calculations in the gas phase of very simplified models of the chemical systems provided qualitative insights on its intrinsic tendency for bond formation, but its energetics could not be directly compared with experimental data. A large energy gain of 50 kcal mol^−1^ for the insertion of a single atom of Mg into the C–Cl bond of CH_3_Cl was calculated using the Hartree–Fock method and MP4 calculations.^38^ Different values should be expected if the solvent effect was included and if the solid nature of Mg was considered. The effect of the solvent was nicely illustrated in a computational study of the Schlenk equilibrium where nonsolvated and solvated (microsolvation) systems were compared.^39^ Without solvent, CH_3_Mg(μ-Cl)_2_MgCH_3_ was calculated to have a free energy 50 kcal mol^−1^ lower than that of the separated CH_3_MgCl monomers. However, in the presence of coordinating Me_2_O, the formation of the dimer is exothermic (by a few kcal mol^−1^) only if entropic effects are included.

### 3.2 Determining the solvation of monomers

More recent computational studies have always introduced solvation effects, usually by combining explicit (microsolvation) and implicit (continuum effects) representations. In agreement with earlier work,^39^ it was shown that the solvation energy increases in the order of Mg(CH_3_)_2_ < Mg(CH_3_)Cl < MgCl_2_. In addition, it was also found that the solvation is stronger with THF than Et_2_O,^40^ a result supported by experiments.^19^ It was also shown that the enthalpy change in the Schlenk equilibrium favours RMg in Et_2_O, but MgR_2_ and MgCl_2_ in THF, which is also consistent with experimental data. Furthermore, it was established that Mg could have more than four groups in its first coordination sphere when polar ligands are attached to Mg. Thus, four solvent molecules (THF) are close to Mg in MgCl_2_, resulting in a distorted octahedral geometry. Other early studies on the structures of Grignard reagents can be found in the review by Yamabe and Yamazaki.^37^

### 3.3 The reaction mechanism pathways

**Fig. 3:**
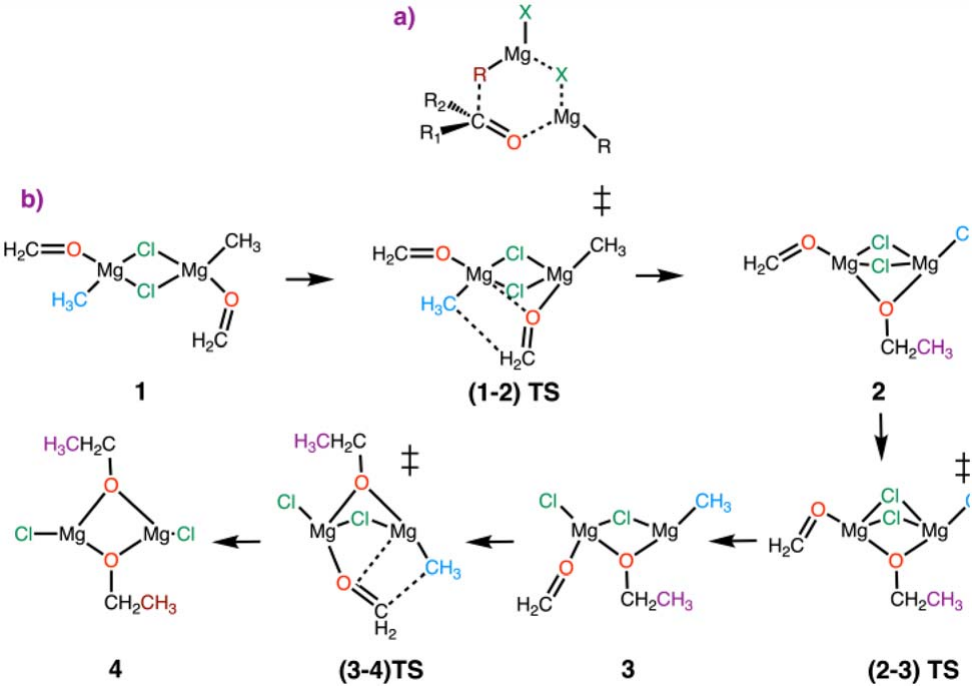
(a) Suggested non-computed 6-membered ring transition state for the Grignard reaction. (b) Reaction mechanism identified by DFT(B3LYP) calculations with CH_3_MgCl and formaldehyde (no solvent) as models. Figure adapted from ref. 37.

There are few computational studies on the Grignard reaction itself. It is interesting to mention a widely quoted six-membered ring mechanism proposed on experimental bases (Fig. 3a),^41,42^ which did not pass computational tests carried out many years later.^43^ What emerged from (DFT)^44,45^ calculations by Yamazaki and Yamabe,^43^ using the B3LYP approximation of the exchange-correlation (xc) functional,^46,47^ was the importance of keeping a dichloro-bridge between the two magnesium centres. The dinuclear magnesium complex and two molecules of substrate were considered as the starting point to include the 1 : 1 stoichiometry between the substrate (formaldehyde) and Grignard reagent (CH_3_MgCl). The reaction mechanism was then studied in the gas phase and with dimethyl ether as solvent. It was shown that the formaldehyde coordinated to a single magnesium reacts preferentially with the methyl group of the other magnesium to form a dichloride-ethoxide-bridged species. This dinuclear species could undergo a further Grignard reaction with the remaining formaldehyde and methyl groups. The complete pathway is shown in Fig. 3b. The energy barriers of these reactions were found to be low, with the first reaction having a lower energy barrier than the second one. It was also found that the coordinating solvent (one Me_2_O per magnesium) did not change the global features, nor did the replacement of Cl by Br.

**Fig. 4:**
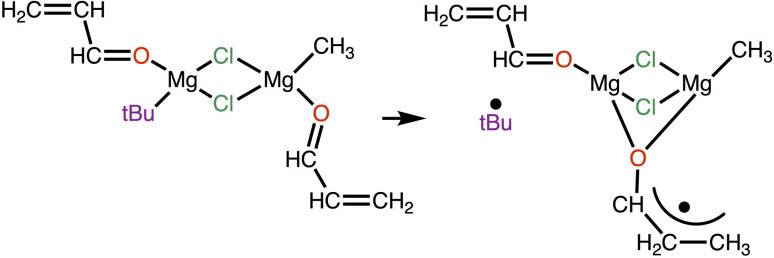
The species involved in the single electron transfer (SET) mechanism of the Grignard reaction, as established by Yamabe and Yamasuki. Figure adapted form ref. 37.

The authors also investigated the SET mechanism. In view of the experiments showing that the SET mechanism is favoured for a conjugated carbonyl as the substrate and a tertiary alkyl R group, the computational model included acrolein as the substrate and a *tert*-butyl group as the R group. Using the dinuclear dichloro-bridged complex previously found to be the preferred reactive system, they discovered that the nucleophilic addition could not be achieved and that an energetically accessible pathway was initiated by the homolytic cleavage of the Mg–C(CH_3_)_3_ bond and the simultaneous displacement of acrolein from a terminal coordination to one magnesium to a bridging position to the two magnesium centres. It was also found that the spin density resulting from the cleavage of the Mg–C(CH_3_)_3_ bond was almost entirely located on the bridging allyl oxide group with almost none remaining at the magnesium centre (Fig. 4).

This study explains some experimental facts but not why the reaction is strongly solvent dependent.^7,48^ It is also likely that species other than the dichloro-bridged dinuclear system are reactive since, for example, dialkyl magnesium itself adds to a carbonyl in the absence of halide delivering species.^49–51^

In another study, the RISM-MP2 method was used to improve the representation of the dynamical electron correlation and the effect of the bulk solvent on the Schlenk equilibrium and the Grignard reaction.^52^ The monomeric species in diethyl ether (2 CH_3_MgCl and Mg(CH_3_)_2_ + MgCl_2_) were found to have a lower free energy than the dimeric forms. The role of the solvent was again found to be important. The same study investigated the nucleophilic pathway of the Grignard reaction. The monomeric species was found to be significantly less reactive than a “linear” dimer with only one bridged chlorine between the two magnesium centres (activation energy: 16.2 kcal mol^−1^ with CH_3_MgCl and 9.2 kcal mol^−1^ with the linear dimer). In the preferred pathway, the substrate (acetone) and the nucleophilic methyl were initially bonded to different magnesium centres.^52^ The solvation was allowed to vary to stabilize intermediates when required.

Despite relevant insights, these studies lacked identification of all the chemical species present in solution and that could react, leaving the open question of the global mechanism for the Grignard reaction, likely more complex than that presented above. This evidence called for the employment of methods that can widely explore the chemical space and correctly describe the thermodynamics of the solute/solvent ensemble.

## 4 The *ab initio* molecular dynamics method

Numerous experimental facts point to an active role of the solvent in the Grignard reaction. In fact, the solvent directly controls the energetics of the Grignard species in solution, mainly through direct coordination to the metal by the ethereal oxygen. The solvent also controls the nuclearity of the Grignard moieties and the reaction rates. Finally, it is chemically intuitive to consider solvent assistance in the ligand redistribution dynamics of the Schlenk equilibrium as well as in the Grignard reaction since these two transformations require the rearrangement of groups ligated to the metals. An accurate investigation of reactive processes involving solvent dynamics/exchange at metal centres requires both a sufficiently good quantum mechanical method to correctly describe the electronic structure of the species and an appropriate treatment of their conformational space, subject to the fluxional dynamics of the solvent. Clearly, this is the realm of *ab initio* molecular dynamics (AIMD).

AIMD takes the ability of molecular dynamics to sample statistically relevant microscopic configurations of a molecular system while maintaining the modelling of the interactions at the quantum mechanical level. Since the breakthrough of AIMD, thanks to the extended Lagrangian formalism by R. Car and M. Parrinello that allowed the on-the-fly propagation of the electronic degrees of freedom^53,54^ and with more recent developments allowing for the direct time evolution of the molecular systems on the Born-Oppenheimer surface,^55–57^ AIMD has been repeatedly used to characterise the structural and dynamical properties of ions in solution. Historically, the most prominent examples have been the characterization of the hydroxyl^58^ and hydronium ions in water.^59^ Over the years, AIMD has helped determine the solvation dynamics of several charged systems, for example, alkali and alkaline earths,^60–63^ transition metals,^64–67^ Al^3+^,^65^ Br^−^,^68^ lanthanides,^69,70^ as well as salts,^71,72^ ionic liquids,^73,74^ and organic and organometallic ionic compounds.^75–79^ Because of the large number of required electronic structure calculations, the typical workhorse for AIMD is DFT. Still today, DFT permits the most advantageous balance between quality and computational costs, even though approaches to go beyond that are now being proposed in the literature.^80^

Despite the clear effectiveness of AIMD in treating these problems, computational studies remained primarily limited to water solutions, ignoring other organic solvents. This is understandable considering the relatively high computational costs of AIMD compared to the molecular complexity of organic solvents, which impose longer simulation times due to slower internal and diffusional dynamics and may require larger simulation boxes to escape finite-size bias. In fact, thanks to the steady increase in computational power, it is now possible to model organic solvent solutions using AIMD. However, another possible source of error in treating conventional organic solvents is related to the fact that the cohesive energy of the liquid is often dominated by weaker interactions (*i.e.*, dispersion forces) than the strong hydrogen-bond network characterising water. Nonetheless, already by using a local GGA functional (PBE) with a dispersion correction, it was possible to predict liquid THF with a density near that of the experiment.^81^ One can expect that other organic solvents can be treated equally well with AIMD, employing DFT functionals that include dispersion interactions in any form.

## 5 What is in the pot?

### 5.1 AIMD studies of CH_3_MgCl in THF

As mentioned above, the diversity of structures representing RMgX is large and also depends on R, X and the experimental conditions such as solvent, temperature, and concentration. Our first study focused on the search for a subset of them, limited to the monomeric and dimeric forms that could be formed from CH_3_MgCl in THF at room temperature, and at a 0.33M concentration.^82^ The solute and solvent were represented equally at the DFT level of theory, using the PBE xc functional,^83^ including empirical dispersion corrections to minimise the bias in the computational exploration of the designed subset.^84^

The key information, which is the solvation of each magnesium, was measured by the coordination number between the Mg centres and the oxygen of THF, defined by the distant-dependent sigmoid function:^85^1
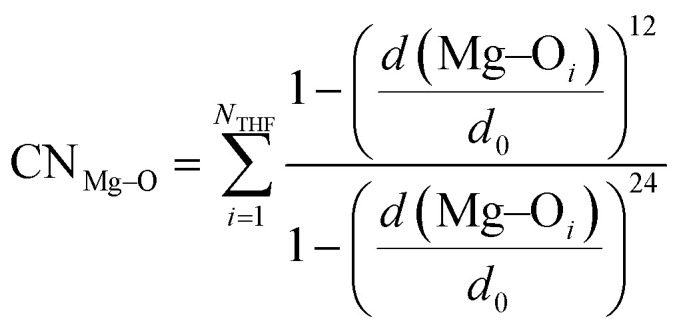
using *d*_0_ = 2.75 Å as the threshold parameter.

**Fig. 5:**
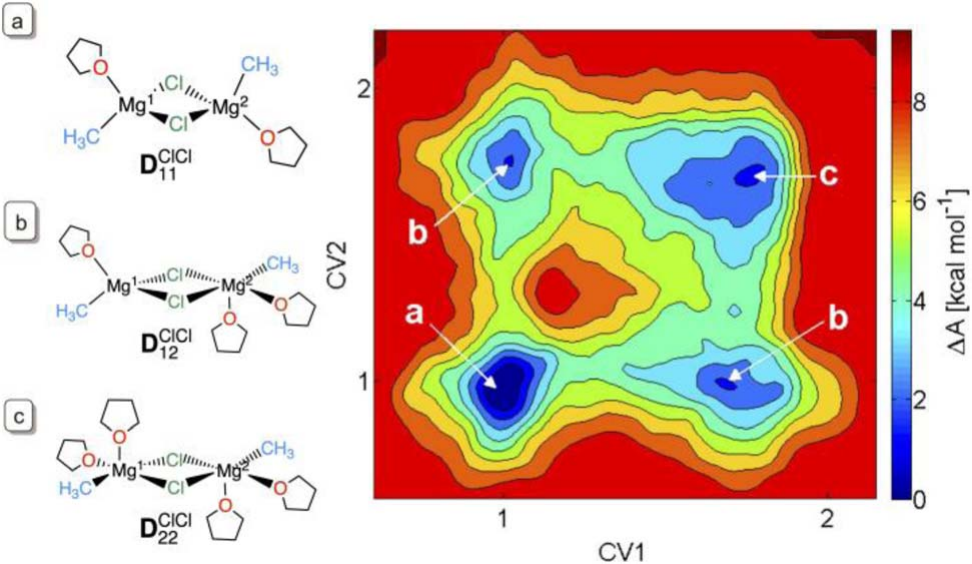
The free energy map of solvation by THF of (CH_3_)Mg(μ-Cl)_2_Mg(CH_3_) by AIMD and metadynamics.^86^ The collective variables (CVs) refer to the coordination number of THF to the two Mg's, according to eqn (1). Panels (a–c) indicate the structures corresponding to the free energy basins. Figure adapted from ref. 82, under ACS AuthorChoice agreement.

For the monomers, calculated data showed that a total coordination of four is favourable for the magnesium atom in all the species, so that (CH_3_MgCl)(THF)_2_, (CH_3_)_2_Mg(THF)_2_ and Cl_2_Mg(THF)_2_ are all plausible structures in solution (Fig. 5). However, for MgCl_2_ only, a higher solvation was found to be preferred with a ratio of 0.07 : 0.1 : 0.01 for the di-, tri- and tetra-solvations. These relative proportions indicate that these differently solvated species are energetically close. The higher solvation of MgCl_2_ is a consequence of the higher polarity of the Mg–Cl bond relative to that of Mg–CH_3_, resulting in a higher positive charge at Mg. Calculations of the structural preference of the dinuclear dichloro-bridged complex (CH_3_)Mg(μ-Cl)_2_Mg(CH_3_) in THF also showed a diversity of solvation modes with similar free energies for one or two THF molecules in the first coordination sphere of each magnesium. There is indeed less than 3 kcal mol^−1^ difference in free energies between (THF)(CH_3_)Mg(μ-Cl)_2_Mg(CH_3_)(THF), D^ClCl^_11_ and (THF)_2_(CH_3_)Mg(μ-Cl)_2_Mg(CH_3_)(THF)_2_, D^ClCl^_22_ (Fig. 5). At room temperature, in THF solution, all coordination modes for all the species can be present, resulting in a highly dynamic first coordination sphere at magnesium.

**Fig. 6:**
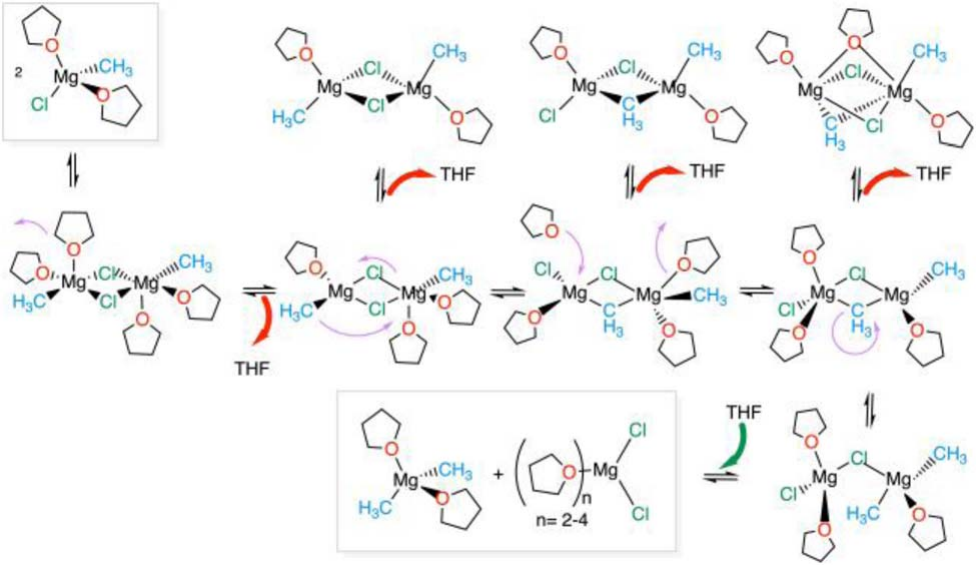
The Schlenk equilibrium and associated solvent dynamics. Red arrows indicate solvent de-coordination, and green arrows indicate solvent coordination. Adapted from ref. 82, under ACS AuthorChoice agreement.

The search for other minima and associated transition states was carried out by studying the CH_3_/Cl exchange pathway for the Schlenk equilibrium using metadynamics,^86^ targeting the solvent coordination number of one Mg, and the difference in coordination of the methyl group on the two Mg centres as collective variables for the transformation.^82^ This led to the identification of a reaction pathway which illustrates how the dynamics of the solvent is essential to assist the passage between minima (Fig. 6). It should be noted that, due to the relatively small size of the simulation box, the determination of the reaction pathway was stopped at a structure where the two methyl groups are coordinated to the same magnesium. This is not the final product of the reaction, which should be the more stable separated MgCl_2_ and Mg(CH_3_)_2_.

The resulting pathway for the Cl/CH_3_ exchange and associated highly dynamic solvation is shown in Fig. 6. The key information from this study is that the solvation at the two magnesium centres must vary during the reaction pathway since the preferred solvation depends on the changeable positions (terminal or bridging) of the chloride and methyl groups. Despite the complexity of the chemical systems, some simple concepts have emerged: (i) it is difficult to move the bridging chloride at maximal solvation (*i.e.* in (THF)_2_(CH_3_)Mg(μ-Cl)_2_Mg(CH_3_)(THF)_2_) and at minimal solvation ((THF)(CH_3_)Mg(μ-Cl)_2_Mg(CH_3_)(THF)) of the two magnesium centres; (ii) the unequal solvation of the two magnesium centres results in a bridging chloride being attracted toward the less solvated magnesium to which it can give more electron density. Thus, the partial over-solvation of one magnesium facilitates the passage of a chloride from the bridging to the terminal position of the less solvated magnesium. Correspondingly, increasing the solvation at one magnesium assists the passage of an R group from a terminal to a bridging position. Thus, the change in solvation at each magnesium facilitates the transfer between the terminal and bridging positions for the chloride and methyl groups, thereby promoting the Schlenk equilibrium. It is worth noting that the most abundant form of the dimers D^ClCl^_11_ is not directly on the Schlenk equilibrium pathway. The less solvated magnesium centres hold tight to the bridging chloride ligands and disfavour any of their displacements.

### 5.2 Variations on the theme: effect of R, X, and the solvent

There is a question regarding to what extent the results obtained for CH_3_MgCl in THF can be generalised, since R, X, and the ether solvent have a strong influence on the behaviour of the Grignard reagent. Another point of interest is the possibility of having in solution aggregates of higher nuclearity than dimers. These points need to be considered to gain a deeper understanding of these challenging species. Quantitative experimental information to validate the computational studies is unfortunately limited. The equilibrium constants 1/*K* of the Schlenk equilibrium (2RMgX *⇌* R_2_Mg + MgX_2_) have been determined for a few Grignard reagents in essentially only two solvents (Table 1, see ref. 40 and reported literature within). It appears that Et_2_O favours RMgX over the redistribution products. This tendency is less pronounced with THF, which favours a more balanced distribution of the three monomers. The influence of R and the halide seems to be globally modest according to the limited data available. Thus, it is essentially the solvent that appears to play a determining role in the equilibrium constant of the R/X exchange.

**Table 1:** Schlenk equilibrium (MgR_2_ + MgX_2_*⇌* 2 RMgX) constant *K* for various Grignard reagents and solvents, obtained using different experimental techniques; data from ref. 40

Grignard	Solvent	Method	*K*
CH_3_MgCl^20^	THF	NMR	1.0 ± 0.72
CH_3_MgCl^87^	THF	IR	4.5
CH_3_MgBr^88^	Et_2_O	Calorimetry	320
CH_3_MgBr^89,90^	Et_2_O	Kinetic + UV	450
CH_3_MgBr^20^	THF	NMR	4.0 ± 2.6
CH_3_MgBr^87^	THF	IR	3.5
EtMgCl^18^	THF	Calorimetry	5.52
EtMgBr^17,91^	Et_2_O	Calorimetry	480
EtMgBr^18^	THF	Calorimetry	5.09
EtMgBr^18^	THF	Calorimetry	1.66
PhMgBr^91^	Et_2_O	Calorimetry	55–62
PhMgBr^18^	THF	NMR	3.77
PhMgBr^92,93^	THF	NMR	4.0 ± 0.8
PhMgBr^94^	THF	NMR	7.46
EtMgBr^95^	MeOCH_2_CH_2_OMe	Polarography	2.2 ± 0.3
PhMgBr^95^	MeOCH_2_CH_2_OMe	Polarography	6.1 ± 0.3

### 5.3 The special case of *tert*-BuMgX

Some data were also obtained for the Grignard reagent with a *tert*-Bu group (Table 2).^20^ At high temperatures, *tert*-BuMgCl is preferred over the redistribution products, while at low temperatures there is a statistical distribution of species. The authors noted that the reaction was considerably slower than with smaller R groups but did not give quantitative data. The experimental conditions are thus important to consider in order to understand the behaviour of all Grignard reagents.

**Table 2:** Temperature dependence of the Schlenk equilibrium constant *K*: *tert*-Bu_2_Mg + MgCl_2_*⇌* 2 *tert*-BuMgCl; data from ref. 20

*T*/°C	*K*
>65	7.54
59	5
51	2.36
42	1.74
33	1.12

## 6 The Grignard reaction: CH_3_MgCl and acetaldehyde in THF

The central questions in understanding the Grignard reaction are (i) which species present in an ethereal solution of Grignard reagent and substrate are reactive and (ii) what favours the radical (SET) mechanism over the nucleophilic addition. Answers to these questions have been recently provided by AIMD and DFT calculations.^96^

### 6.1 The nucleophilic addition

#### 6.1.1 A mechanism with many pathways

In the early study described above,^37^ a dinuclear magnesium complex was identified as being particularly reactive. The structure of the transition state was hardly modified by the solvent coordinating at the magnesium centres. Similarly, the activation energy was not significantly affected by the presence of solvent in the initial and transition states. However, we saw that the solvent dynamics could be fundamental.^82^ For this reason, we decided to use AIMD to study the reactivity of both monomers and dimers. As there are many candidates in each category and no reason to exclude anyone, we considered studying them all.

**Fig. 7:**
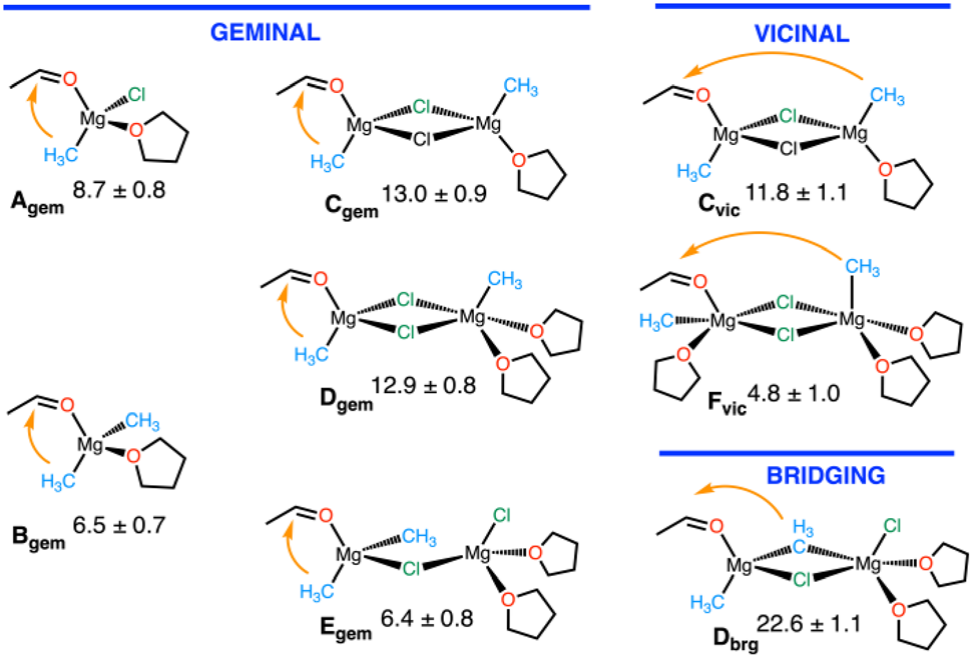
List of monomers and dimers considered as reagents and associated free energy of activation in kcal mol^−1^, as determined by thermodynamic integration. Adapted from ref. 96, under CC-BY-NC-ND 4.0 license.

The only hypothesis implemented in the study was that, in order for the reaction to proceed, the substrate had to be coordinated to a magnesium centre. How the substrate enters the coordination sphere of the magnesium (putatively, by ligand exchange with one solvent molecule) was not explicitly investigated. Considering all the locally stable structures identified during the study of the Schlenk equilibrium, we obtained an initial pool of possible reactant structures as shown in Fig. 7. In the monomers, the substrate and the nucleophilic methyl group are coordinated to the same magnesium (geminal position). In the dimeric species, the substrate and the nucleophilic methyl group can be either on the same (geminal), or on different (vicinal) magnesium atoms. It can also be at a bridging position.

**Fig. 8:**
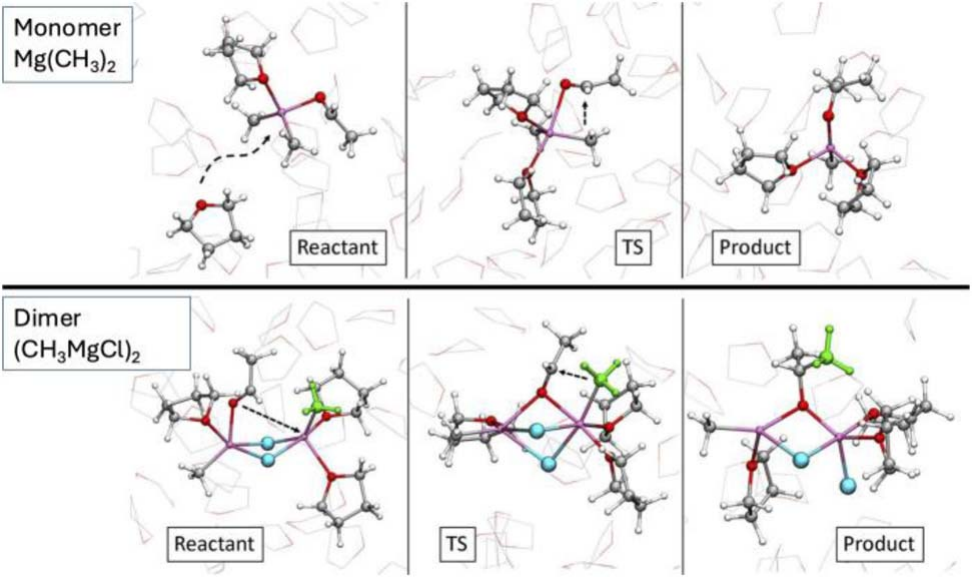
Comparison of the reaction mechanisms for monomeric (B_gem_) and dimeric (F_vic_) complexes. In the first case, the reaction is helped by an incoming solvent molecules coordinating Mg at the TS. In the second case, the initial higher coordination of the metal centres allows reaching the TS without any major reorganisation of the environment. Figure adapted from ref. 96, under CC-BY-NC-ND 4.0 license.

The activation energy for the addition of the methyl group to the acetaldehyde carbon to form a magnesium-bound isopropyl alkoxide ligand was determined for each of these species. All five geminal reactions induce a four-centre transition state (C=O–Mg–C_Me_) which creates an electronic vacancy at the Mg centre associated with the cleavage of the Mg–methyl bond. Solvent is, therefore, required to fill the empty coordination site and stabilise the magnesium species. Thus, all geminal reactions, on either a monomeric or a dimeric species, require the participation of a solvent that enters the magnesium first coordination sphere at the appropriate moment of the reaction pathway (usually at the transition state or immediately before). There is no report of this pathway in any previous study because the methods were not adapted to model a solvent addition during a reaction. The activation barriers for these four-centre geminal transition states are relatively low, ranging from 6.5 to 13 kcal mol^−1^. The small differences in energy are easily understood by the way the ancillary ligands manipulate the nucleophilicity of the methyl group and by the rigidity of the molecule. Thus, the activation barrier is lower for Mg(CH_3_)_2_ (B_gem_) than for Mg(CH_3_)(Cl) (A_gem_) because the ancillary CH_3_ group is more electron donating than Cl, as evidenced by NBO analysis,^82^ making the other methyl group more nucleophilic. E_gem_ has the same reactivity as B_gem_ since Mg has two methyl ligands and a fourth ligand which is the chlorine of a di-solvated MgCl_2_ group. This fourth ligand has a similar influence on the reactive Mg as a coordinated solvent (B_gem_). The lower reactivity of C_gem_ and D_gem_ can be understood by noting that the two bridging chlorine ligands are weaker electron donors to the reactive Mg and rigidify the coordination at the magnesium, disfavouring the formation of a four-centre transition state requiring a small bond angle (O_Ac_–Mg–CH_3_). C_gem_ is apparently too rigid to allow a similar transition state, resulting in a higher activation barrier. In Fig. 8, we illustrate, for the case of B_gem_ how a nearby well-oriented THF solvent molecule enters the magnesium coordination sphere at the transition state, facilitating its development into the final product.

The vicinal reactions also occur *via* a four-centre type transition state, obtained by translocation of the carbonyl group from a peripheral to a bridging position. However, the solvent contribution is different from that in the geminal reactions. F_vic_ is associated with the lowest action energy of all species, while interestingly C_vic_ which differs only by the degree of solvation from F_vic_ is associated with one of the highest activation energies. In the latter case, the rigidity of the minimally solvated dichloro bridged magnesium dimer is likely to be the reason for the high activation energy as it was for C_gem_. The major difference between the vicinal and geminal reactions is that in the former one, the two magnesium centres are involved in the electrophilic activation of the acetaldehyde since the ligand is bound to both of them in the transition state. In addition, this bridged activated acetaldehyde is in close proximity to the nucleophilic methyl group. Interestingly, the two chlorine atoms remain bridged to the two magnesium atoms, which also helps to keep the free energy of this transition state low. Another factor that helps to prevent an unfavourable decrease in entropy is that this highly solvated structure does not need the assistance of any additional incoming solvent molecule. The reason for this is that the substrate remains bonded to its “original” magnesium and creates a new bond to the other magnesium. No empty coordination site is created at either magnesium during the formation of the alkoxy group. The reaction pathway for this most favourable reaction is illustrated in Fig. 8 by the representation of three snapshots for the reactant, transition state and product.

Finally, as expected, D_brg_ has a very high activation since it is evident that a bridging methyl group is a less good nucleophile than a terminal one. It should be noted that our study identifies a dimeric complex as the most reactive species, as did the study of Yamazaki and Yamabe.^43^ However, the coordination mode of the substrate (formaldehyde in the prior study) in the transition state is different in the two studies. In particular, in the former study, the formaldehyde was found to be bound to only one magnesium centre at the transition state, and the influence of solvent on the nature of intermediates and transition states was not considered.^43^ More importantly, the earlier study identified a single possible pathway, whereas the new study shows that many transition states, starting with monomeric and dimeric reagents, are close enough in energy to be considered as competing nucleophilic pathways occurring in parallel under standard experimental conditions.

#### 6.1.2 Structural features of the transition states in the geminal and vicinal pathways

In the 1970s, crystal structures were used to determine the geometrical features of the nucleophilic addition to a carbonyl group.^97^ The examination of the crystal structure data was completed with *ab initio* Hartree–Fock calculations using H^−^ as the nucleophile and H_2_CO as the substrate, all in the gas phase due to the notorious computational limitations of the time.^98^ Both studies showed that the nucleophile, Nu, approaches the carbonyl carbon with a Nu⋯C–O angle larger than 90° (angle between 105 and 110°, known as the Bürgi–Dunitz angle). This direction of approach optimises the overlap between the nucleophile HOMO and the substrate LUMO 
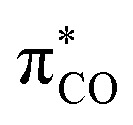
. This finding was at the heart of the rationalisation of the stereoselectivity of the nucleophilic addition, known as the Felkin–Anh rule.^99–101^ It should be noted that these early studies of selectivity only considered the nucleophile and ignored the accompanying metal, which is essential to enhance the electrophilicity of the substrate. Thus, it is of interest to determine whether the Bürgi–Dunitz angle is also found in the AIMD computational study of the addition of CH_3_MgCl to acetaldehyde. The calculations found that it was indeed the case for the two preferred pathways (geminal and vicinal). This result extends the range of validity of the Bürgi–Dunitz angle.

### 6.2 SET mechanism

Experiments have indicated that a SET mechanism could compete with, or be preferred to, the nucleophilic mechanism. The SET mechanism was suggested to involve the formation of R^˙^ radicals by homolytic cleavage of the Mg–R bond. However, quantum (DFT) static calculations give the bond dissociation energy (BDE) of Mg–CH_3_ in CH_3_MgCl(THF) (*i.e.* formation of H_3_C^˙^ and ClMg^˙^) as high as 66 kcal mol^−1^. This value, in very good agreement with the experimental value of 61 kcal mol^−1^ obtained for CH_3_MgBr in diethyl ether,^102^ remains high even when increasing Mg solvation.^96^ Such a high BDE is incompatible with a reaction running at room temperature or even lower. Evidently, this discrepancy does not originate from the computational method of choice but from the chemical model selected to probe this mechanism. Indeed, there is no report of Grignard reagents producing radical species in the absence of a substrate. This suggests that the substrate has a direct role in selecting a SET mechanism. Therefore, it needs to be included in the calculations of the homolytic cleavage of the Mg–R bond. The H_3_C–Mg BDE was thus calculated in (CH_3_)Mg(Cl)(substrate)(THF)_*n*_ (*n* = 1, 2) where the substrates considered are shown in Table 3.^96^ Test calculations showed that similar values were obtained with Mg(CH_3_)_2_.

**Table 3:** Calculated magnesium-methyl carbon homolytic bond dissociation energy in (H_3_C)–Mg(Cl)(substrate)(THF)_*n*_, at the DFT level of theory, using the PBE XC functional. Values are in kcal mol^−1^. Data from ref. 96

Substrate	BDE (*n* = 1)	BDE (*n* = 2)
CH_3_CHO	51	38
CH_2_O	45	33
CF_2_O	35	25
Fluorenone	29	16

**Fig. 9:**
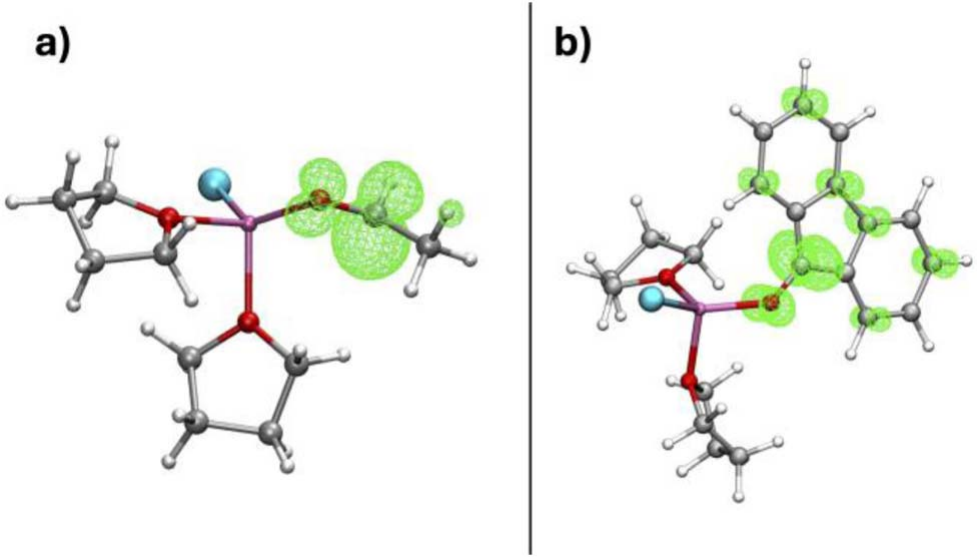
Spin density localization on (a) ^˙^Mg(Cl)(acetaldehyde)(THF)_2_ and (b) ^˙^Mg(Cl)(fluorenone)(THF)_2_. Adapted from ref. 96, under CC-BY-NC-ND 4.0 license.

The coordinated substrate considerably lowers the Mg–CH_3_ BDE. This effect can be rationalised by noting that, in the ^˙^Mg(Cl)(substrate)(THF)_*n*_ radical, the unpaired electron localises at the LUMO 
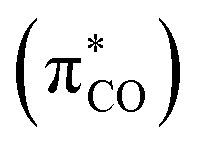
 of the coordinated substrate (Fig. 9), while, in its absence, it remains on the magnesium. This electron localisation was also obtained by Yamazaki and Yamabe.^43^ The role of the substrate in assisting the Mg–R BDE is thus clear. Substrates with a low reduction potential (*i.e.* low-lying 
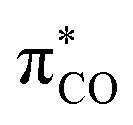
 LUMO) stabilize the magnesium radical and thus lower the BDE, as illustrated by the decreasing values in the order of acetaldehyde, formaldehyde, difluoroketone, and fluorenone (Table 3). Other conjugated ketones like benzophenone should also give low Mg–R BDEs. This rationalizes the observation of a SET mechanism for conjugated carbonyls.^31,103^ While the SET mechanism is favoured by the presence of the low-lying empty orbital of the conjugated substrate, the nucleophilic pathway is found to be disfavoured by this substrate. The bulk of the conjugated substituents at the carbonyl carbon hinders the structural rearrangement of the coordination sphere at the magnesium required during the reaction pathway. This was shown by AIMD calculations for Mg(CH_3_)_2_ and fluorenone.^96^ This may also explain why, all things being equal, *tert*-BuMgX is associated with a greater tendency to proceed *via* a SET pathway. These results are in agreement with the experiments. As mentioned in the introduction, experimental data have established that alkyl ketones do not go *via* the SET mechanism and prefer the nucleophilic pathway and that often the nucleophilic and SET pathways are both possible even with conjugated carbonyl species.^34,35^

In our opinion, the most important message emerging from these calculations is the identification of a manifold of accessible pathways for the Grignard reaction. Many magnesium complexes are competent for a diversity of nucleophilic mechanisms. Furthermore, in the case of conjugated carbonyl substrates, the nucleophilic and SET pathways can also have similar activation barriers. The nature of the Grignard reagent, substrate and solvent also influence the relative preference between the possible pathways, but it is unlikely that experimental conditions result in a strong preference for a single pathway. This explains the difficulty in determining the reaction order. Additionally, this also accounts for the challenge of rationalising the mechanism of this reaction for the ensemble of possible chemical species involved. Finally, this introduces new perspectives on the way one should approach reaction mechanisms in Mg chemistry.

## 7 Beyond the Grignard reaction

### 7.1 The key role of lithium salts

Grignard used only magnesium derivatives for his reaction. It had been an incredible breakthrough at that time, even though the process was not very efficient, not so easy to handle, and not particularly selective. The choice of magnesium happened to be a very good compromise between the poorly reactive zinc dialkyl^3^ and the very reactive and hard to control calcium derivatives. Over the years, significant improvements have come from combining Grignard reagents with other metal derivatives.^104–106^ Mastering the reactivity of calcium is also now becoming possible, and recent studies have begun exploring the viability of strontium.^107–111^ Here, we focus on the first great improvement of the Grignard reaction, achieved by Paul Knochel by combining Mg and Li species, named the turbo Grignard because of its high efficiency.^112,113^

The addition of LiCl to the reactive media improved all aspects of the reaction and, in particular, allowed Cl/Mg exchange between haloarenes, Ar-X, and Grignard reagents. This was very useful for the *in situ* synthesis of a wide variety of functionalized arylmagnesium and heteroaryl Grignard reagents, Ar-MgX, from the reaction of iPrMgX (X = Cl, Br) with a wide variety of Ar–X.^112^ The reason why lithium chloride speeds up the reaction^105^ is of topical interest and is still essentially not understood, although proposals have been made based on DFT calculations.^113,114^ Both studies propose associations of the Grignard reagent, LiCl and substrate, and analyse the reactivity of these ensembles with appropriate DFT methodology. Unfortunately, the existence of the proposed species in solution under *operando* reaction conditions needs further analysis. Indeed, any interpretation of the reactivity of the turbo Grignard should include an analysis of the accessibility of postulated structures formed by the association of RMgX and LiCl in organic solvent, for which, unfortunately, related experimental or theoretical studies are scarce.

Crystalline species were isolated from a solution of Grignard reagent and LiCl. Remarkably, no mixed Mg/Li species were characterised and, as it was already noted for the case of Grignard reagent alone, the isolated crystalline species^115^ may not be present in solution. Conflicting results have also been reported. For instance, NMR studies suggested that Mg/Li association is possible, whereas electrospray ionisation mass spectrometry (ESI MS) study revealed the formation of Mg only species with various numbers of Mg. These contrasting results highlight the influence of the concentration (higher in droplets than in the reactive media) on the nature of possible complexes.^28^ The challenging question of the nature of the RMgX·LiCl complex in solution also requires a fundamental understanding of the nature of LiCl alone in the relevant organic solvent. This question was recently approached by us in two ways. In a first study, using AIMD, we provided a description of the solvation structure of LiCl in THF and also determined its affinity with the Mg species involved in the Schlenk equilibrium. More recently, exploiting AIMD data, we calibrated a machine-learning interaction potential to carry out a comprehensive study of the chemical space for LiX (X = Cl, Br and I) in THF.

### 7.2 Simulating LiCl in THF and its interaction with CH_3_MgCl

Lithium halides tend to exhibit weak solubility in organic solvents,^116,117^ giving rise to the current opinion that their solubilised particles should anyway retain a certain degree of aggregation, with the diamond ring (LiCl)_2_ representing an evident possible building block for higher-order structures. Consequently, a primary pertinent inquiry was to utilise AIMD to ascertain whether solvated (LiCl)_2_(THF)_4_ would persist as a dimer or exhibit a tendency to form aggregates of higher nuclearity in THF solution, as they do in the solid state. Indeed, AIMD calculations showed that (LiCl)_2_(THF)_4_ dimers, set at a relatively long distance, spontaneously form a tetramer without suffering any activation barrier during the partial desolvation.^118^ This tetramer evolves toward a pseudo-cubane type species with a geometry similar to that observed in the solid state. However, AIMD simulations suggest that, at room temperature, this pseudo-cubane has high plasticity with a preference for a broken Li–Cl edge of the cube. We deduced from this result that this open edge could promote the interaction with magnesium species through the formation of Mg–Cl and/or Li–Cl bonds between (LiCl)_4_ and magnesium derivatives.

**Fig. 10:**
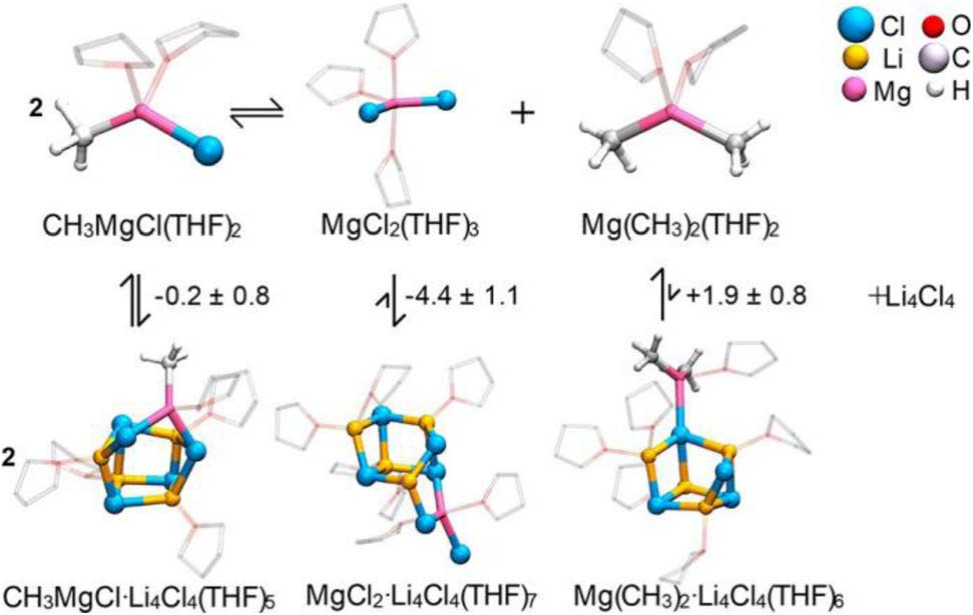
Interaction of the monomeric forms of the Grignard reagent with a (LiCl)_4_ cluster. Free energies are in kcal mol^−1^. Figure adapted from ref. 118, under CC-BY 4.0 license.

Specifically, our study investigated its interaction with CH_3_MgCl, Mg(CH_3_)_2_ and MgCl_2_ – the endpoints of the Schlenk equilibrium. Among these three forms, only MgCl_2_ binds to (LiCl)_4_ to form MgCl_2_·(LiCl)_4_. In contrast, CH_3_MgCl has a marginal affinity to (LiCl)_4_, and Mg(CH_3_)_2_ prefers to stay away from the lithium-chloride cluster. Thus, the preferred binding of (LiCl)_4_ to MgCl_2_ pushes the Schlenk equilibrium towards two homoleptic forms, decreasing the concentration of the standard CH_3_MgX species (Fig. 10).^118^

**Fig. 11:**
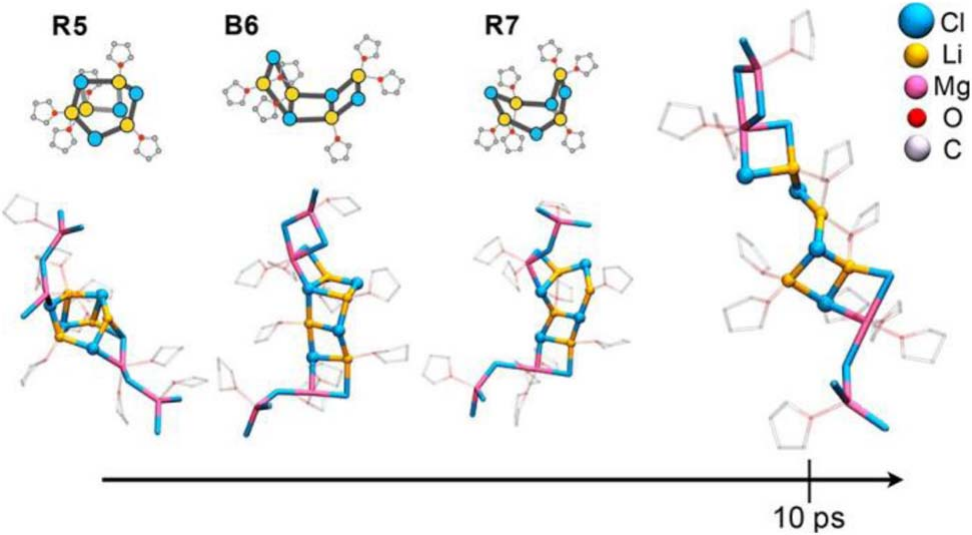
Time relaxation of solvated (LiCl)_4_ interacting with MgCl_2_ in a 1 : 1 Mg : Li ratio. Figure adapted from ref. 118, under CC-BY 4.0 license.

An analysis of the charge density in this mixed Li/Mg chloride aggregate indicates an electron density shift from (LiCl) to MgCl_2_, suggesting that MgCl_2_ could tend to disaggregate the LiCl cluster. To probe this further, we studied a 1 : 1 concentration of LiCl and MgCl_2_ in THF, analogous to Li : Mg stoichiometric ratios used in turbo-Grignard solutions. AIMD data show that the LiCl cluster evolves by successive cleavage of LiCl bonds and opening of the (LiCl)_2_ rhombus. Within 10 ps, we found an essentially decomposed (LiCl)_4_ cluster with MgCl_2_ coordinated on the outside of the unstructured lithium chloride moiety. There, almost all of the closed (LiCl)_2_ units had transformed into linear moieties or mixed LiMgCl_2_ rings (Fig. 11).^118^ Although we are not able to substantiate this hypothesis yet, we can speculate that this decomposed LiCl cluster could better interact with the substrate than the original, rather insoluble, compact LiCl entities.

This preliminary study^118^ revealed a complex dynamical aspect for LiCl in THF, in the presence of Grignard reagents. MgCl_2_ can contribute to the formation of more dispersed, more accessible, more soluble and therefore more reactive LiCl entities. This could facilitate the interaction between the substrate and LiCl. However, the reactive entities remain undefined. MgR_2_ should be present in abundance, relative to RMgX, and dimers of RMgX should be disfavoured due to the possible shift of the Schlenk equilibrium. Thus, the remaining lingering question is: what are the reactive species?

In the study of the reaction of Grignard with a carbonyl function, we have shown that several forms of Grignard reagents have similar reactivity and in particular that a monomer and a dimer are among the most reactive forms. It is likely that the reactivity with other substrates, particularly haloarene, should follow a similar pattern. These are directions to consider for a better understanding of the reactivity of the turbo Grignard, especially its best representative, iPrMgX·LiCl, in the X/Mg exchange reaction with haloarenes.

Before attempting this, we felt it was essential to have a better insight into the large chemical space of lithium halides in THF. Clearly, structural characterization of LiX in solution, and worse in the presence of Grignard compounds, is a combinatorial puzzle composed of a large number of ways to assemble a number of Li–X, and Li–O bonds. A thorough exploration of such a vast configurational space is a substantial challenge for a computationally expensive method like AIMD, even empowered by enhanced sampling techniques. On the other hand, quantum chemical accuracy is required in order to take into account different bond polarization energies that may occur in the presence of a varying number of more or less strong electron-donating groups at the alkali centres. The necessity of accurate energies and energy gradient, and efficient conformational sampling methods is today realised by the advent of machine-learning interaction potentials. Here, we first describe the method before exploring its application to determine the structure of Li-X (X = Cl, Br and I) in THF.

## 8 Machine-learning potentials: a brief introduction

**Fig. 12:**
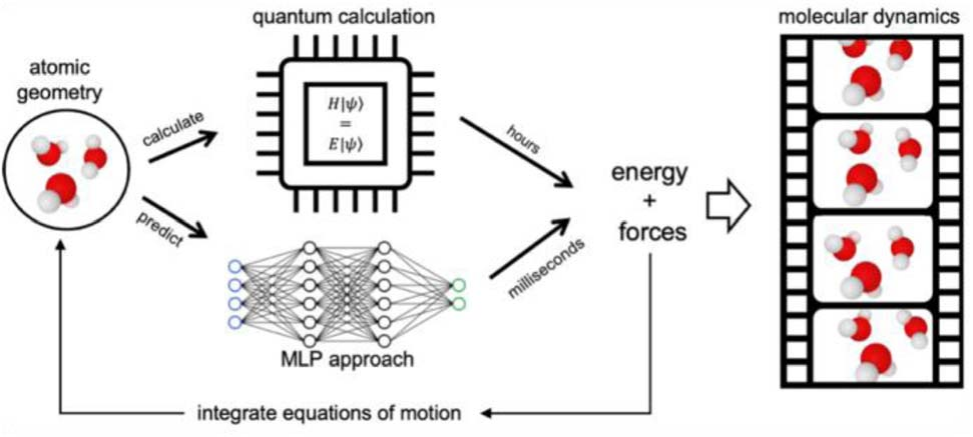
*Ab initio vs.* MLP approach for generating molecular dynamics sampling.

AIMD using DFT and plane-waves,^53^ which is among the fastest *ab initio* approaches, can typically target systems involving hundreds to few thousand atoms on timescales of tens to hundreds of picoseconds. These time and size constraints limit which type of phenomenon can be targeted for mechanistic investigations. More and more, the field is shifting away from *ab initio* to machine learning potential (MLP)-based MD. The central idea is illustrated in Fig. 12. To do MD, we need the forces for a molecular geometry to integrate the equations of motion. In AIMD, this is done by numerically solving the Schrödinger equation, which is computationally costly. In MLP-based MD, the forces of the same *ab initio* potential energy surface are instead predicted by a neural network-based MLP powered by GPUs, which is orders of magnitude faster. As such, MLP-based MD can break the time and size constraints of traditional *ab initio* approaches.

In MLPs, the local geometric environment serves as the primary input, typically consisting of atomic species and the distance vectors to neighbouring atoms within a predefined cut-off radius. This geometric information is used to feed a neural network, which subsequently outputs predictions for energy and forces. The task of determining the model parameters in an MLP can be formulated as a regression problem:

2 *y*_i_ = *f*(*x*_i_,*β*),

where the model parameters, (*β*), are optimized using training data that consist of geometries, (*x*_i_), to closely approximate reference energies and forces, (*y*_i_), derived from *ab initio* calculations. By using combinations of distance vectors, MLPs can represent many-body interactions. By combining this with a huge set of MLP parameters, the intricacies of the true quantum mechanical interactions can be faithfully represented.

However, MLP-based MD offers no free lunch. First, the use of an MLP introduces the additional step of having to develop a model, requiring the construction of a training set composed of molecular geometries and associated energy and forces from quantum chemical calculations and model training. Second, the MLP is only, in principle, as good as the *ab initio* approach used in constructing the training set, and, in practice, worse, due to reliability issues stemming from an inability to fully generalize from training set geometries to MD sampled geometries. Ways to reduce computational costs for training MLP potentials, and increase their reliability, are today turning around three main concepts: (i) active learning (AL) targeting reaction coordinates of interest; (ii) moving from invariant to equivariant MLPs; (iii) foundational models and fine-tuning.

While previously gathered *ab initio* MD data can provide a solid foundation for the training set, an MLP naively trained on such data is typically unreliable and prone to catastrophic failure due to gaps in the MLP surface. The critical concept of AL involves systematically constructing the training set based on molecular geometries collected from different iterations of MLPs. Typically, these new MD geometries are gathered using MLP-based MD, supplemented by uncertainty estimation to add only those geometries that the MLP poorly describes. Through repeated iterations, this process reduces the difference in molecular geometries encountered during MLP-based MD and the training set data, leading to reliable MLPs.

Mechanistic studies typically target rare events along specific reaction coordinates. Not having sufficient data on the characteristic geometries for such events in the training set and relying on the deduced MLPs to predict them is, at best, a hit-or-miss and can, at worst, lead to incorrect mechanisms. To address this issue, Parrinello and coworkers incorporated enhanced sampling of the reaction coordinates as an integral part of the AL process, as exemplified in their seminal study on urea decomposition.^119^ Similarly, van Speybroeck and coworkers have been developing impressive modular AL workflows that emphasize enhanced sampling.^120^ Perego and Bonati have created a code for AL of MLPs that are uniformly accurate along the reaction coordinates, applying it to the study of ammonia decomposition over iron–cobalt alloy catalysts.^121^ Although it introduces added complexity, integrating enhanced sampling simulations with AL offers an excellent opportunity for more agile science, where hypotheses about mechanisms can be refined throughout the AL process.

The reliability of neural-network-based MLPs hinges not only on the quality of the training data but also fundamentally on the neural network architecture. Early on, the importance of incorporating symmetries was recognized.^122^ Crucially, the MLP architecture must account for the invariance of molecular energy concerning translations, rotations, and permutations of identical chemical species. This understanding led to the development of the seminal Behler–Parrinello network, which leverages symmetry functions to ensure these invariances are respected.^122^ By employing invariant features such as interatomic distances and angles as inputs, the Behler–Parrinello network effectively captures the inherent symmetries of molecular systems, thereby enhancing the reliability and robustness of the models.

However, while implementations of MLPs based on invariant features, such as DeePMD and ANI,^123^ are state-of-the-art in terms of computational efficiency, the field is progressively shifting away such architectures. In particular, it has been found that using non-scalar inputs, such as distance vectors or spherical harmonics, and having the neural networks transform internal features equivariantly. For instance, when a molecule's geometry *x* is rotated, it transforms as *R*·*x*, where *R* is the rotation operation. Similarly, equivariant neural networks incorporate layers *f* that ensure internal features transform equivalently, maintaining the relationship *f*(*R*·*x*) = *R*·*f*(*x*). This leads to MLPs that are more accurate and need less data for training. For example, NequIP, developed by the Kozinsky group, can achieve higher test set accuracy with 1000× fewer data than DeePMD for the water phase-diagram.^124^ There is also mounting evidence that such equivariant MLPs are better at avoiding catastrophic model failures,^125^ which is a frequent problem during the early iterations of AL. Moreover, methods and software are constantly being improved; implementations such as PaiNN,^126^ NequIP,^124^ Allegro,^127^ and MACE^128^ have matured to the point where they are frequently being adopted for mechanistic studies.

As MLP technology improves, there is also a push to handle more complex tasks. One of the most active areas of research is developing general-purpose MLPs, often called foundational models, which aim to represent any chemical system. These models use similar architectures but typically include more parameters and are trained on vast amounts of data collected over years of computational chemistry research. While this ambition might have once seemed futuristic, recent progress shows that these foundational models are maturing.^129,130^ In particular, a community effort has led to the development of a MACE-based foundational model built on data from the Materials Project. This model demonstrated an ability to represent various chemical systems, including solids, liquids, gases, chemical reactions, interfaces, and even small proteins.^131^

While these advancements are impressive, the use of foundational models in mechanistic studies is still in its early stages and they are not yet ready to be deployed out of the box. However, in the short term, these foundational models offer significant value as the basis for pre-trained or fine-tuned MLPs. This approach, common in machine learning, involves initializing the model parameters with those from another model previously trained on a different dataset. By starting from a foundational model, an excellent starting point is established that leverages pre-learned representations and patterns. This pre-training process accelerates convergence, enhances predictive performance, and reduces the quantity of new data required for training. Furthermore, since less training data is needed, higher quality quantum chemistry data, such as coupled cluster calculations, can be employed to potentially mitigate one of the critical weaknesses of MLPs—being only as good, or worse, than their low-quality training set data. Consequently, this approach allows for the efficient adaptation of MLPs to specific mechanistic studies, facilitating more robust and accurate simulations in complex molecular systems. For excellent examples of how this can be implemented in practice, we suggest that readers refer to ref. 132 and 133.

## 9 MLPs and main group chemistry – first steps

### 9.1 Lithium halide polymorphism: experimental data

**Fig. 13:**
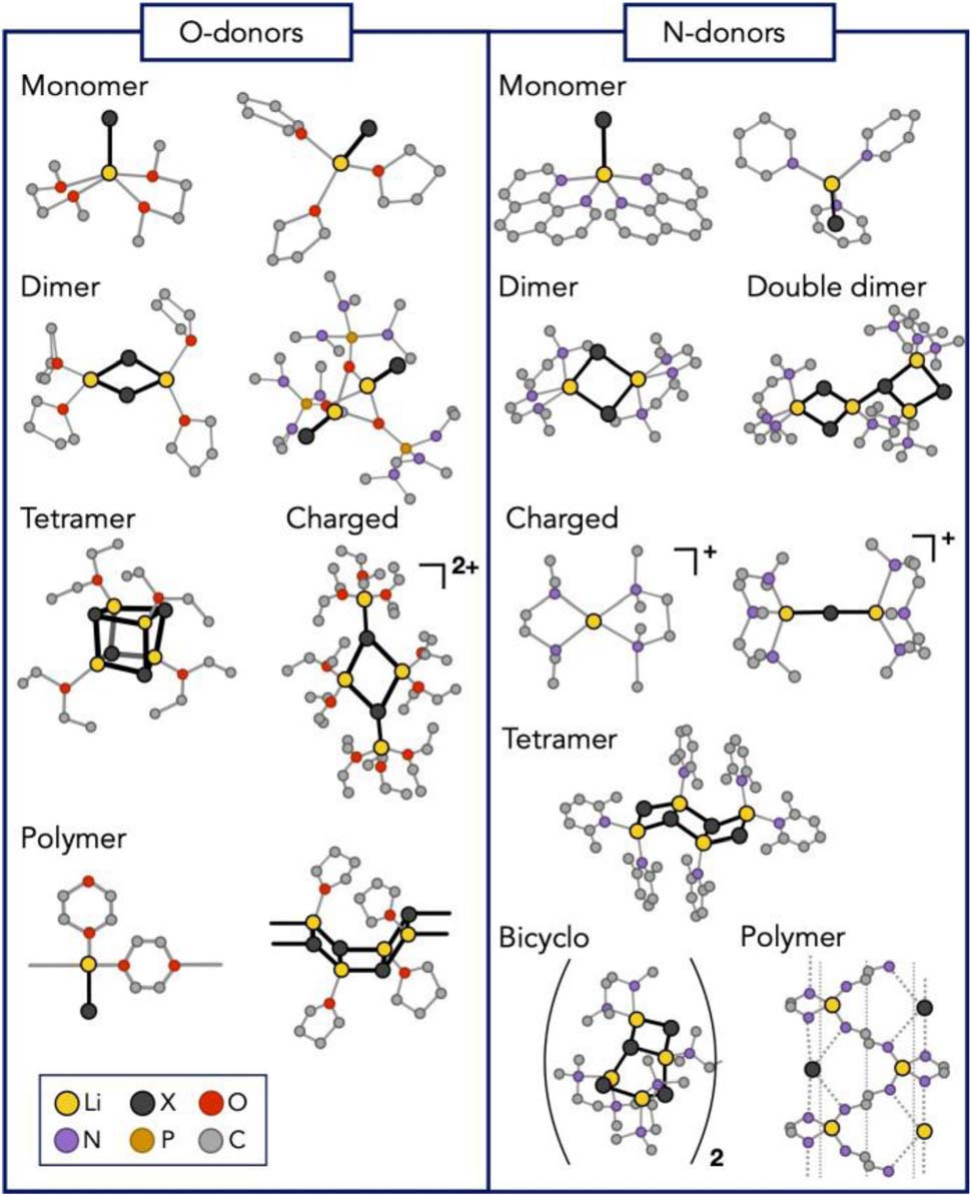
Isolated and structurally characterized lithium halide complexes. See de Giovanetti *et al.* (2024) and references within.^81^ Figure adapted from ref. 81, under CC-BY 3.0 license.

Numerous LiX aggregates have been isolated from organic solution through coordination with diverse O- and N-Lewis bases and characterised by X-ray diffraction studies. A representative but not comprehensive list of structures is shown in Fig. 13 The structures are highly diverse in nuclearity and shapes and include *inter alia*, dimers, tetramers, clusters, oligomers, and polymers (see ref. 81 and references within). Such a rich ensemble of structures raises the following questions. Are all these species (and eventually other ones) also present simultaneously in THF solution, and in what relative abundance, or are the structurally characterised species simply driven by the Lewis bases and the ease with which crystalline phases can be formed?

The number of experimental studies of lithium halides in solution is relatively limited and the information somewhat contradictory. ^7^Li NMR suggests that LiCl exists as a dimer in THF and that disaggregation to monomers can be induced by the addition of excess hexamethylphosphoramide.^134^ In contrast, LiBr would prefer a monomeric structure, whereas LiI would exist as a mixture of contact ion pairs, monomers and dimers.^134^ However, in another study using lithium NMR, it was concluded that all lithium halides (Cl, Br, and I) would prefer to be a solvent-separated ion pair.^135^ LiBr was also reported to be a cubic tetramer in toluene.^136^ Unfortunately, these experiments were conducted with low concentrations of lithium halide to minimise signal broadening, which does not reflect the experimental conditions under which these salts are used. Furthermore, the somewhat contradictory results may reflect the very high sensitivity of these systems to experimental conditions. This is therefore an interesting challenge for a molecular modelling study. For the reasons described earlier, a machine learning potential approach was selected. With this MLP method, it was possible to study broadly the three lithium halides LiCl, LiBr and LiI in THF. The extensive validation of the MLP and the procedures used are described in the original article.^81^

### 9.2 Computational study of lithium chloride

**Fig. 14:**
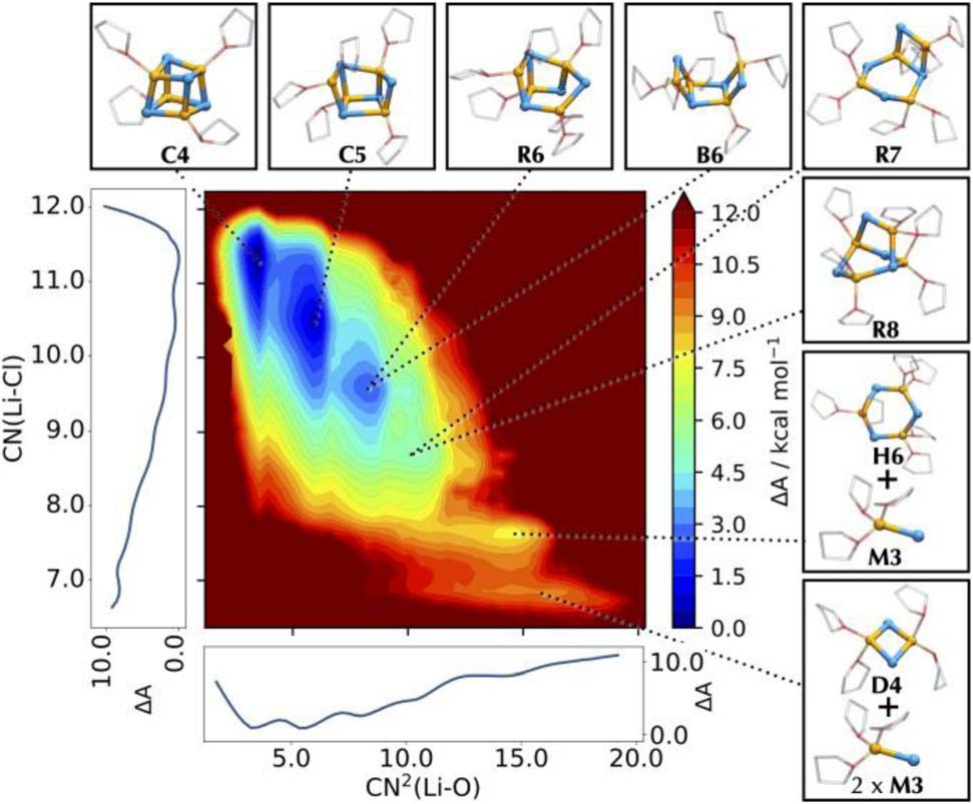
Helmholtz free energy surface of the conformational landscape of (LiCl)_4_, using an MLP. The coordination number of Cl around Li, CN(Li–Cl), and the squared coordination number of O around Li, CN^2^(Li–O), are used as collective variables for the FES. Side graphs report the projections of the FES onto two respective axes. Representative structures corresponding to the basins of the FES are shown as balls-and-sticks (Li in yellow, Cl in cyan, and THF as transparent sticks). Figure adapted from ref. 81, under CC-BY 3.0 licence.

The Helmholtz free energy map is projected in two dimensions using lithium solvation (CN^2^(Li–O)) and the cluster compactness (CN(Li–Cl)) as collective variables (Fig. 14). Using these indicators, highly clustered, poorly solvated structures (such as pseudo-cubane) appear at low CN^2^(Li–O) and high CN(Li–Cl). The free energy surface in Fig. 14 shows the presence of a large number of structures at similar energies. The pseudo-cubane, C4, or edge-opened pseudo-cubane, C5, are among the most stable species. As the lithium solvation increases, the LiCl cubic cluster further degrades by cleaving more Li–Cl bonds. Different structures appear, depending on the number and positions of the Li–Cl bonds, at slightly higher energies relative to the most stable C4 and C5. Species with (LiCl)_2_ rhombi are preferred over rings of diverse sizes. Many of these structures (*i.e.* C4, B6, R7, D4, *etc.*) correspond to species that have been isolated out of solution (see Fig. 13). Solvated monomers together with dimers appear only at higher energies. Thus, the isolation of dimers and monomers in the solid state is likely to be promoted by the interaction with powerful Lewis bases that are able to stabilize smaller clusters. This confirms that LiCl would not easily form monomers or dimers without the assistance of additional chemical species. This is compatible with the above presentation of the deconstruction of (LiCl)_4_ by MgCl_2_.^118^

Given that LiCl is often used at a quasi-saturated concentration (1 M for an experimental solubility of around 1.27 M in THF), we also investigated the effect of increasing LiCl concentration on the preferred structures in solution. MLP simulations showed that separate pseudo-cubane units merge exothermically, and with a low energy barrier, to form hexagonal-packed structures (wurtzite-like lattices) that are likely to be good starting particles towards the nucleation into the rock salt structure of solid LiCl, similarly to what was previously reported for aqueous solutions of NaCl.^137^

### 9.3 Lithium bromide and lithium iodide

The free energy surface for (LiBr)_4_ is similar to that for the chloride case, but there is a clear tendency towards a preference for more solvated and less compact structures.^81^ The more stable structures are no longer the most compact pseudo-cubanes species but distorted variants with one or two broken Li–Br bonds. The solvated dimers are more accessible than LiCl. The behaviour of LiI extends the trends already visible with the bromide – compact structures, notably the pseudo-cubanes, do not appear among the preferred structures, and, in contrast, dimers and monomers become thermodynamically favoured.

### 9.4 Why these trends?

These calculated trends are compatible with the increased solubility of LiX in the order Cl < Br < I.^116,117^ Even though there was no attempt to quantify solubilisation in our study, it is interesting to note that the tendency for the clusters to break down into smaller units for heavier halides is compatible with the solubility trends. This compatibility between the FES and the solubility trends further confirms the validity of the MLP calculations.

These results are easily understood from the polarity of the Li–X bonds as a function of X and the shape of the aggregates. The Li–X bond is more polar for lighter halides, as it was indicated by the NBO charges.^81^ Similarly, the Li–X bond is more polar when there are fewer X atoms bonded to each Li and fewer Li bonded to each X. Therefore, the heterolytic cleavage of a Li–X bond with the aid of a neutral incoming coordinated solvent (THF) requires less energy for the less polar bonds. Thus, the pseudo-cubane (LiX)_4_ is easier to cleave with heavier halides, and the ease of cleavage decreases as the clusters become smaller. This makes monomer formation energetically difficult, and more so for Cl than I. The increase in the ionic character of Li–X as the size of the aggregates decreases has been experimentally established in the case of Li–C and Li–N bonds.^138^ The similarity between Li–C and Li–Cl ionic character has been suggested to be a reason for the halide contamination in lithium alkyl species.^139^

## 10 And now what, looking ahead?

### 10.1 When is AIMD important?

Successes and challenges of computational methods for studying the mechanisms and reactivities of chemical species in solution have been the subject of perspectives and reviews.^140–143^ Historically, AIMD approaches have often been considered as computationally expensive and have often been disregarded in favour of more efficient static, implicit solvent models. In fact, such static approaches commit to intrinsic approximations in the representation of temperature effects, in the dynamics of the solute–solvent interactions, and in the appropriate ensemble averaging. This leads to difficulties in representing the physics of the experimental systems and their thermodynamics. AIMD has been able to overcome several of these limitations,^144–149^ especially for systems where conventional models are likely to introduce biases that could lead to significant errors. Not surprisingly, AIMD has been used to study reactions where the solvent itself is either a reagent or an essential partner. The (incomplete) list of reactions comprises systems in water,^149–155^ or protic solvent (ref. 156 and references therein), reactions of dihydrogenation, dihydrogen transfer, and hydrogen evolution,^157–160^ water splitting or oxidation,^161–165^ and reactions with several limiting pathways.^166^

The need for dynamic approaches becomes more and more pressing as the strength of the ligand field decreases. This may be the case for main-group elements (Groups I and II), characterised by fluxional structures with labile coordination shells and considerable solvent/ligand exchange, as well as elements from Group X to at least Group XIII.^167,168^ For compounds containing these elements, structures are often flexible and sensitive to the environment, and a method like AIMD can be useful or even mandatory. It is remarkable that one of the first applications of AIMD in organometallic chemistry concerns the study of organolithium derivatives.^169^ This study of exotic dilithioethylene, carried out at that time in the absence of solvent, already highlighted the great diversity of ways in which lithium can interact with a chemical group. Lanthanide complexes in solution also benefit from an AIMD simulation, as shown in a recent publication.^170^

### 10.2 Delving into Group I and II chemistry

Focusing on Group I and II metals, organolithium species have an important role in numerous reactions.^171^ The nature of the bonding between the metal centre and main group elements has been for long time a topic of interest, as illustrated by the recent experimental and computational study of Li–C and Li–N bonds.^138^ Characterization of complexes in the solid state^172–175^ was complemented by NMR measurements to determine the structure of aggregates in solution.^176^

Our recent experience with LiX points to the need for a thorough exploration of the conformational space, in order to fully account for the diversity of species in solution, which can be achieved by integrating *ab initio* calculations with MLPs. The use of MLPs to describe main group chemistry is further supported by the evidence of their ability to address fundamental chemical transformations, such as ammonia decomposition at lithium imide surfaces, as recently shown by Parrinello and co-workers.^177^ It is easy to predict an increased effort in this direction, benefitting also from the rapid methodological development aimed at the definition of more and more efficient protocols for the calibration of MLPs and from the introduction of numerically more robust neural networks.

A question relevant to this perspective is whether AIMD methods and in particular the use of MLPs would be useful to better describe the chemical reactions of highly complex species such as those of Group I and II elements and mixtures of them. We have already shown in this perspective how little was known about the nature of the compounds and especially the reactive compounds under *operando* conditions. If a few years ago, AIMD studies of the Grignard species were limited to various forms of monomers and dimers,^96^ recently, ML acceleration has allowed investigation of the chemical spaces of lithium halides from monomers to pre-nucleating crystalline forms.^81^ This opens up the possibility of going much further, for example, delving into reactive mixtures of RMgX and LiX, and its next step – adding a substrate to the mixed system and study the prototypical X/Mg exchange reactions enabled by this turbo Grignard reagent. This approach could establish the origin of its efficiency while avoid making speculative hypotheses on the nature of the Mg, Li and Mg/Li species present in the solvent.^112,114^

Worthy of further computational exploration with MLPs are the nature and reactivity of lithium alkyls, lithium amides and related species for which interesting experimental information has been obtained. Lithium complexes have been studied in particular by lithium NMR, which gives valuable information about their nature under conditions similar to the *operando* conditions. They have also been studied by various computational methods usually involving dynamic approaches but with limited exploration of the chemical spaces. Combination of AIMD simulations and NMR showed that ^1^*J*_Li,C_ coupling is mostly sensitive to the degree of aggregation and the first coordination shell.^178^ Dynamic QM/MM studies showed that lithium preferred tetracoordination and also indicated large Li–C distance fluctuation, suggesting an easy access to open structures.^179^ Mixed LiR/LiOR tetramers were shown to be fluxional.^180^ AIMD studies of (LiCH_3_)_*n*_ (*n* = 1 – 8) in the gas phase informed on intrinsic Li–C bonding properties of the clusters in the absence of solvent. Thus, tetramers and hexamers are preferred, and temperature plays an important role in the structural preference.^181^ The challenging mixed aggregates nBuLi/RLi (R = Me, *n*-BuO) were studied by Li NMR using a new tool to perform ^7^Li–^7^Li scalar coupling and static calculations together with selected AIMD simulations.^182^ MeLi/LiCl exchange in a lithium amide/LiCl aggregate was shown to occur through an edge-to-edge interaction between the R_2_NLi/MeLi aggregate and (LiCl)_2_ by NMR and AIMD simulations.^183^ The remarkable proposal that only dimers (MeLi)(LiCl) and no tetramers exist in a mixture of methyllithium and lithium chloride in THF has been suggested by NMR measurements and static calculations.^184^ The protective role of lithium chloride against the hydrolysis of lithium amide was suggested by mass spectroscopic measurements and static DFT calculations.^185^

One obvious strategy to make exploration as broad and complete as possible, while being compatible with computational time, is to drastically reduce the cost of computations, especially the electronic calculation part. We have successfully explored this possibility in the case of lithium halides with the machine learning potential. However, a machine learning potential is specific to each problem, which raises the question of its transferability. This needs to be further explored, as the degree of synergy that could result from constructing MLPs for complex mixtures from those developed for each individual subset of species (*e.g.*, RMgX with LiY from separated RMgX and LiY) needs to be established. If it works, this could facilitate the simulation of the whole experimental system, including reagents, precatalysts, and additives from the modeling of the individual parts. In Group I and II chemistry, this would be helpful for the exploration of heterometallic systems and would provide insight into the observed synergy resulting from the addition of additives (such as lithium salt).^105,185^ It will also help to investigate the polar organometallic chemistry in bio-based solvents and water,^186^ which is largely computationally unexplored.^187^

### 10.3 Integrative modelling – a possible way forward

The studies of lithium chemistry presented in the paragraph above explored a relatively limited part of the conformational space. However, simulations were validated by the available experimental data, in particular by use of NMR measurements. Matching calculations with experiment is a productive way to validate the relevance of the structures considered in a study. This is nicely evidenced by quantitative calculations of the NMR data, which indeed require inclusion of conformational dynamics to accurately reproduce the experimental values.^188,189^ Nevertheless, the size of the combined chemical and conformational spaces to be explored can easily become gigantic and increase dramatically with each new species of interest added to the analysis. It is quite clear that a complete exploration is essentially beyond the scope of computational facilities and that chemical knowledge would and should still be a guide to exploring the most relevant parts of the chemical space in particular depth. However, one should not hesitate to explore outside the “expected” zones of significance where new data could probably be discovered and verified by quantitative calculations of any physical property. An illustrative area is that of the allowed and forbidden pericyclic reactions which have essentially initiated the field of computational studies of reaction mechanisms with semi-empirical methods and are now being studied quantitatively.^190^ It should be noted, however, that in these studies the reagents are well identified so that the improvements could focus on the accurate description of pathways of different electronic nature (concerted, zwitterionic, diradical, *etc.*). The difficulty is thus considerably higher when the chemical species are not known at a molecular level.

The computational costly part is associated with the calculations of the electronic structures and atomic forces. DFT with a preference for non-hybrid functionals was thus selected as a method of choice for the electronic structure calculations. So far, ML studies using post-HF wave-function-based methods are limited. However, they did show promising results in topics where non-bonded interactions are essential, such as for inert molecules physisorbed or chemisorbed in porous materials,^191^ as well as in strongly correlated systems like actinides under radiation,^192^ zero field splittings for lanthanide complexes,^193^ molecular collisions,^194^ photodissociation issues,^195^ and computations of EPR parameters.^196^

One possible way forward comes from the integration of relatively fast and accurate computational methods, such as MLPs, with experimental data in a synergistic strategy. The possibility of driving molecular simulations, for example, by imposing geometric restraints from NMR data, is an old idea, mainly explored in the field of structural biology. Today, this original rough idea is empowered by the ability to combine on-the-fly calculations of any property with MD and to use the discrepancy between the predicted and experimental signals to drive the exploration of the conformational space towards more representative structures. Typically, the discrepancy is defined by a loss function to be minimised, such as the square deviations between the two signals. The instantaneous bias force can be introduced considering the likelihood that the instantaneous geometry explored during MD belongs to the ensemble of structures populating the experimental sample by Bayesian statistics. This inference method, taking the name Metainference,^197^ has been recently introduced for the determination of thermally accessible conformational ensembles in soft/polymeric systems, prominently proteins and surfactants, matching all-atom MD to 2D ^1^H and ^15^N NMR,^197^ as well as to small-angle scattering data.^198,199^

**Fig. 15:**
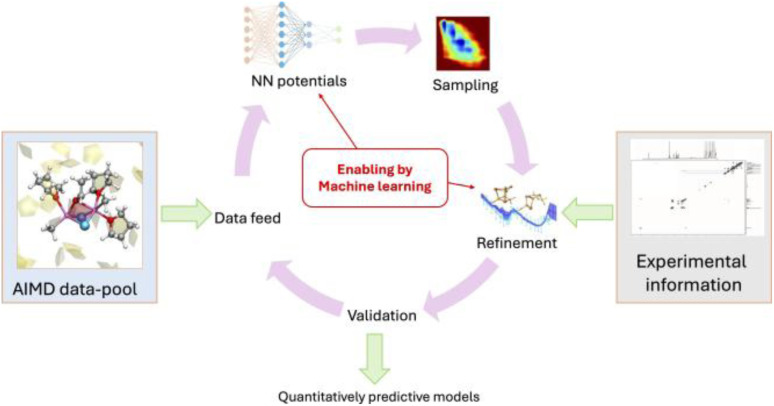
Possible workflow for a computational model incorporating the development of a machine learning potential from *ab initio* data, further refined over experimental insights by exploiting data-driven approaches, to achieve quantitative agreement with experiment and high predictivity.

Metainference, as well as other types of integrative data-driven models, promises to provide a way toward quantitative agreement between simulation and experiment (Fig. 15). In its general formalism, any quantity that can be inferred from structural data, as well as any combination of multiple quantities, can be used as a target. For example, one of us has recently used metainference to simultaneously address independent data from small-angle X-ray and small-angle neutron scattering experiments to reveal the polymorphic nature of the self-assembly of non-ionic surfactants.^200^ The extension of this methodology to reveal the detailed nature of chemical, and in particular organometallic compounds has not been attempted yet, but all the technical elements are now in place.

Possible elements of risk relate to the nature of the signal for these systems, which, confined to NMR, to avoid an excessive broadening of the peaks, may be recorded at significantly lower temperatures than the relevant *operando* conditions. For highly fluxional systems such as main-group compounds, this may lead to a bias in the structural diversity of the sample being observed. From a computational perspective, a significant complicating factor can be the need of establishing sufficiently accurate, and at the same time computationally non-demanding, on-the-fly quantum-mechanical methods for determining chemical shifts or *J* couplings, which may be particularly sensitive to the local fluctuations in both the geometric structure and the solvent.^188,189,201^ Possible ways around this difficulty, without sacrificing the quality of the predicted properties of interest, may again be provided by machine learning approaches, for example, by exploiting the active-learning sampling procedure to acquire information not only on the energy and forces associated with a given geometry but also on specific properties of interest, which may prominently include NMR signals.^202–206^

### 10.4 What one could hope to do

By establishing protocols and computational methods that are practical and accurate for exploring the high complexity of multi-component multi-step chemical transformations, one may be able to identify the nature and role of each species that swims in the large and often obscure pool of solution chemistry. This is a desirable extension of our earlier study in which we attempted to identify the nature of the LiX species present in a pool of THF.^81^ These methodologies would hopefully give the keys to enter the challenging and still poorly explored world of chemical species whose behaviour and reactivity are strongly influenced by the solvent and the experimental conditions. In this way, the chemistry of many metal species, which has been challenging for decades, could be approached computationally with greater chance to give a fuller image of “what is going on”.

## Data availability

No primary research results, software or code has been included, and no new data were generated or analysed as part of this perspective.

## Author contributions

MC and OE proposed the topics and discussed the contents of various sections. OE wrote the main body of the text. SLB wrote the section dedicated to MLPs. MC contributed to the writing in all the sections.

## Conflicts of interest

There are no conflicts to declare.
